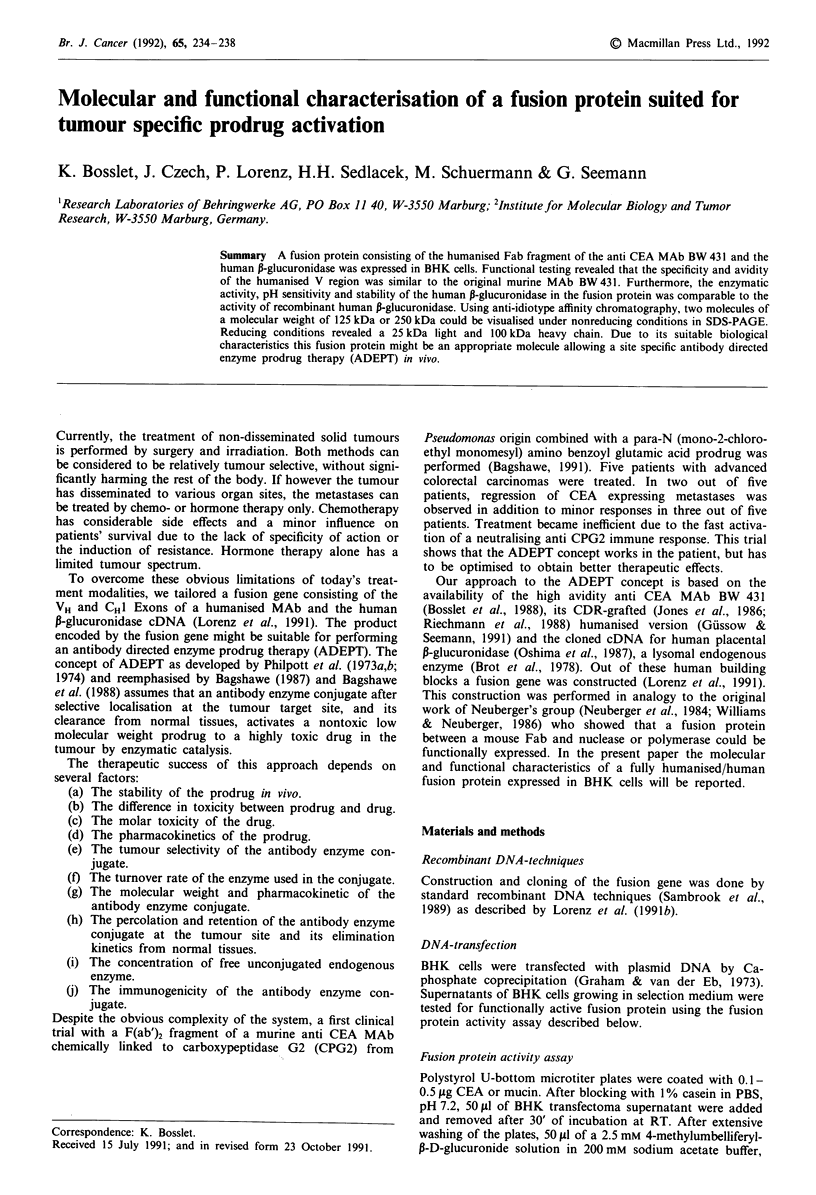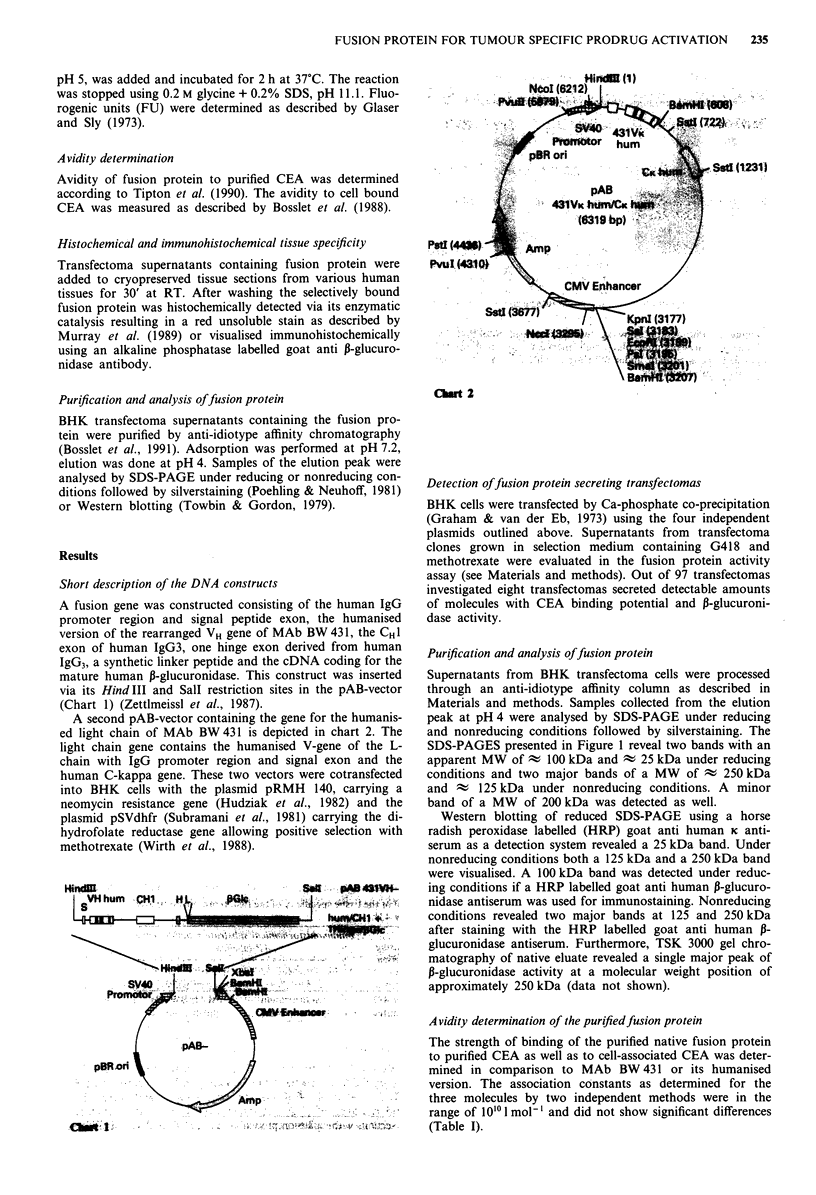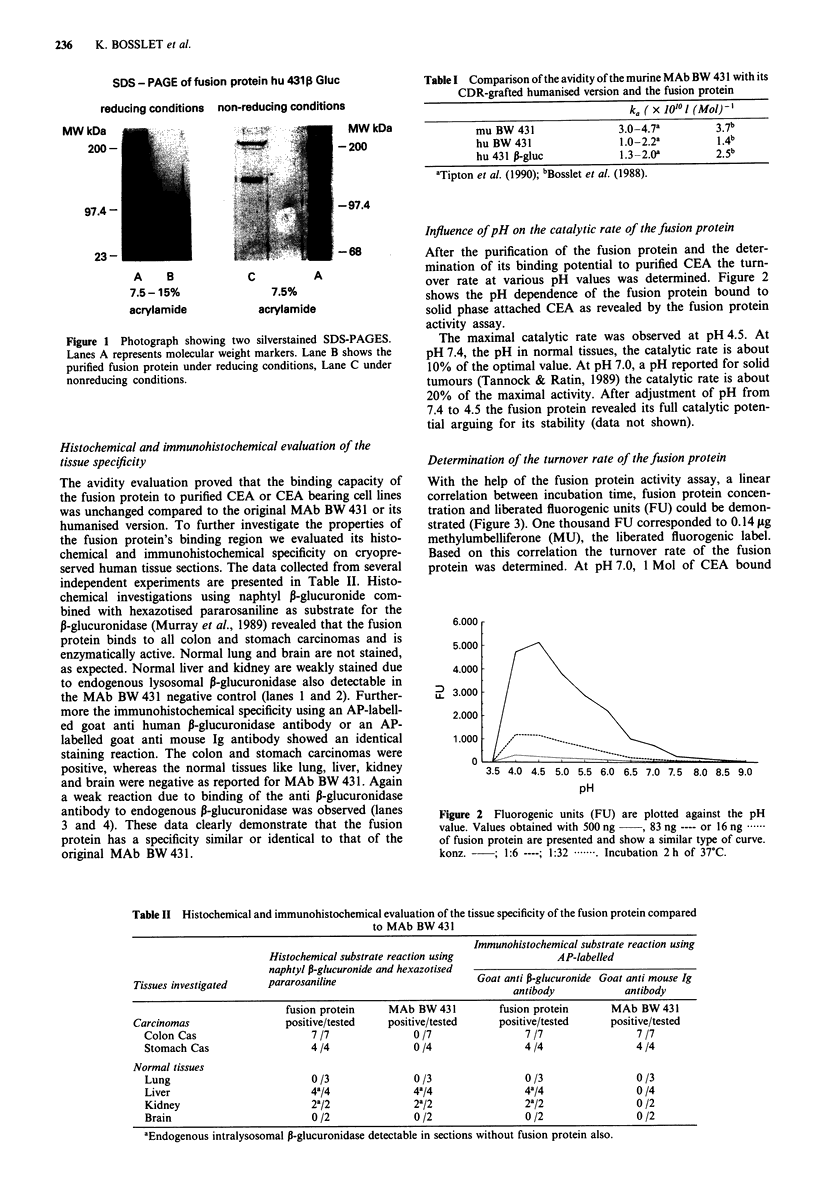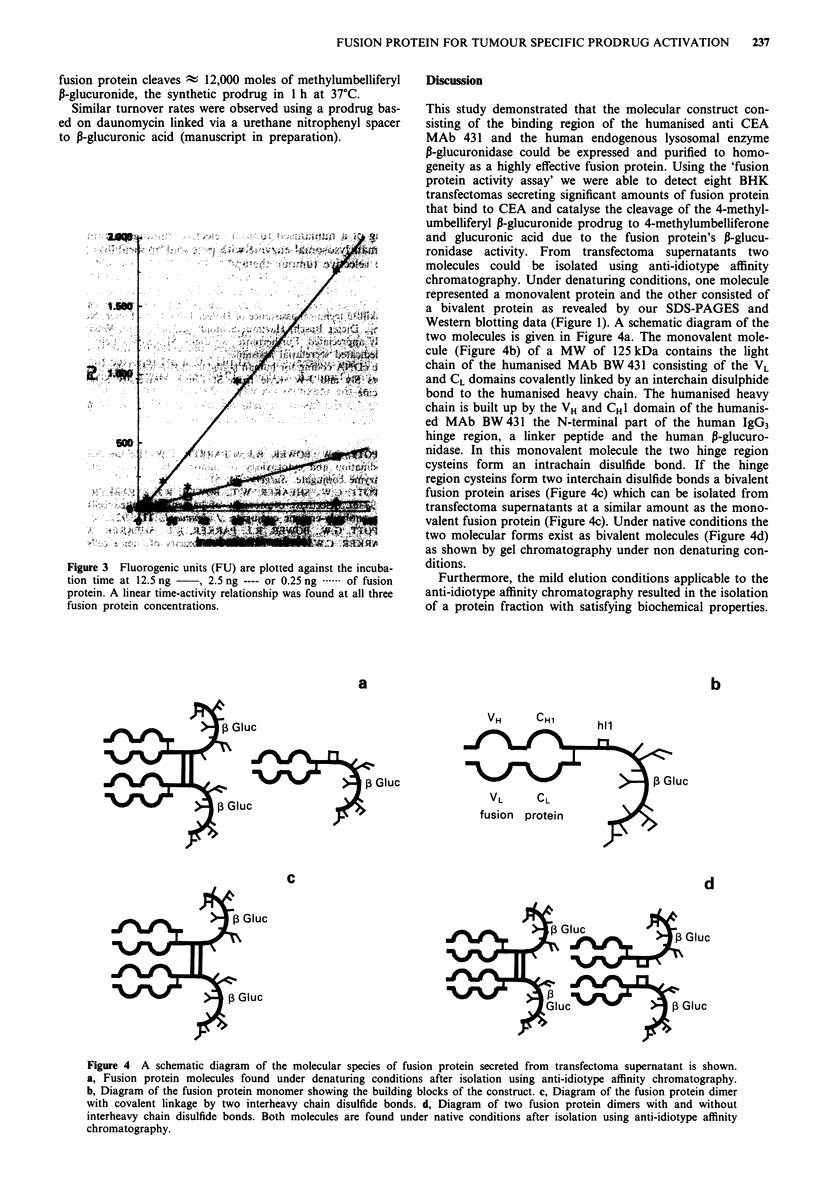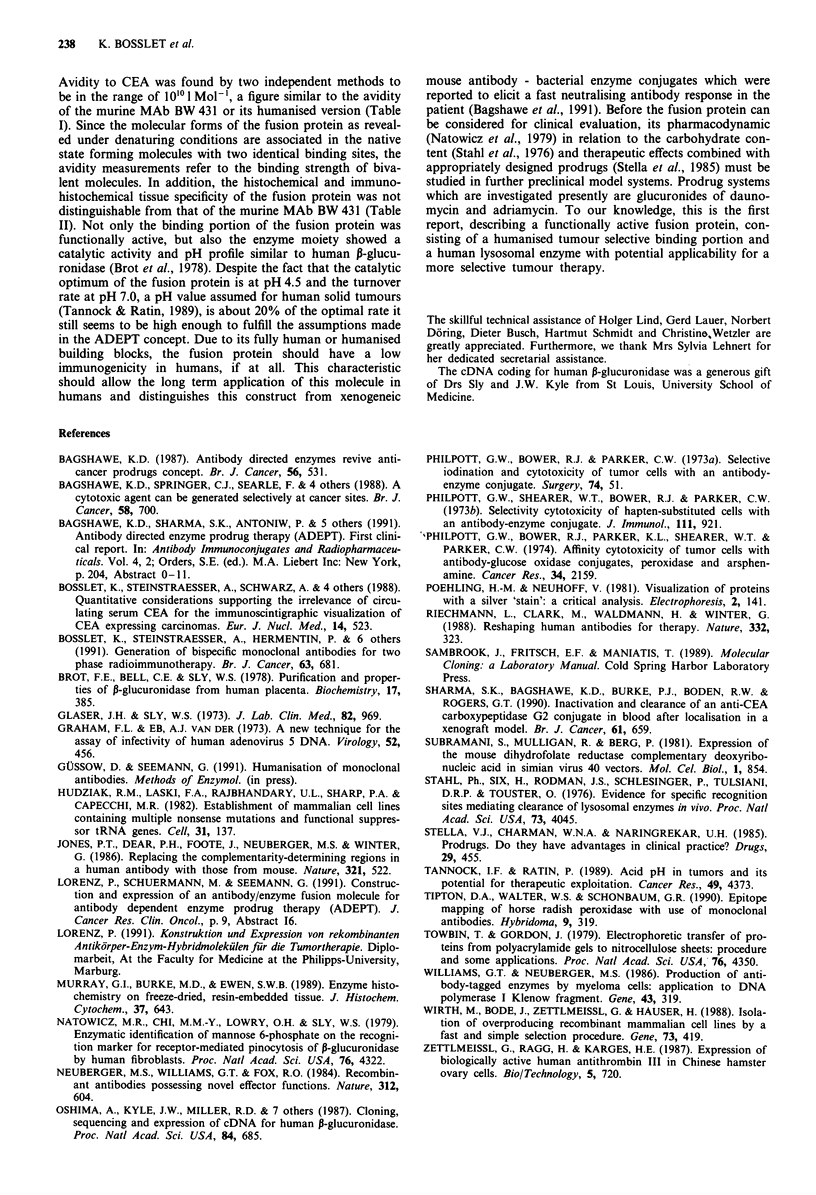# Molecular and functional characterisation of a fusion protein suited for tumour specific prodrug activation.

**DOI:** 10.1038/bjc.1992.47

**Published:** 1992-02

**Authors:** K. Bosslet, J. Czech, P. Lorenz, H. H. Sedlacek, M. Schuermann, G. Seemann

**Affiliations:** Research Laboratory of Behringwerke, Marburg, Germany.

## Abstract

**Images:**


Br. J. Cancer (1992), 65, 234-238 © Macmillan Press Ltd., 1992

### Molecular and functional characterisation of a fusion protein suited for tumour specific prodrug activation

K. Bosslet, J. Czech, P. Lorenz, H.H. Sedlacek, M. Schuermann & G. Seemann

[1]*Research Laboratories of Behringwerke AG, PO Box 11 40, W-3550 Marburg;* [2]*Institute for Molecular Biology and Tumor Research, W-3550 Marburg, Germany.*

**Summary** A fusion protein consisting of the humanised Fab fragment of the anti CEA MAb BW 431 and the human β-glucuronidase was expressed in BHK cells. Functional testing revealed that the specificity and avidity of the humanised V region was similar to the original murine MAb BW 431. Furthermore, the enzymatic activity, pH sensitivity and stability of the human β-glucuronidase in the fusion protein was comparable to the activity of recombinant human β-glucuronidase. Using anti-idiotype affinity chromatography, two molecules of a molecular weight of 125 kDa or 250 kDa could be visualised under nonreducing conditions in SDS-PAGE. Reducing conditions revealed a 25 kDa light and 100 kDa heavy chain. Due to its suitable biological characteristics this fusion protein might be an appropriate molecule allowing a site specific antibody directed enzyme prodrug therapy (ADEPT) *in vivo*.

Currently, the treatment of non-disseminated solid tumours is performed by surgery and irradiation. Both methods can be considered to be relatively tumour selective, without significantly harming the rest of the body. If however the tumour has disseminated to various organ sites, the metastases can be treated by chemo- or hormone therapy only. Chemotherapy has considerable side effects and a minor influence on patients' survival due to the lack of specificity of action or the induction of resistance. Hormone therapy alone has a limited tumour spectrum.

To overcome these obvious limitations of today's treatment modalities, we tailored a fusion gene consisting of the $V_H$ and $C_H1$ Exons of a humanised MAb and the human β-glucuronidase cDNA (Lorenz *et al.*, 1991). The product encoded by the fusion gene might be suitable for performing an antibody directed enzyme prodrug therapy (ADEPT). The concept of ADEPT as developed by Philpott *et al.* (1973*a*,*b*; 1974) and reemphasised by Bagshawe (1987) and Bagshawe *et al.* (1988) assumes that an antibody enzyme conjugate after selective localisation at the tumour target site, and its clearance from normal tissues, activates a nontoxic low molecular weight prodrug to a highly toxic drug in the tumour by enzymatic catalysis.

The therapeutic success of this approach depends on several factors:

(a) The stability of the prodrug *in vivo*.
(b) The difference in toxicity between prodrug and drug.
(c) The molar toxicity of the drug.
(d) The pharmacokinetics of the prodrug.
(e) The tumour selectivity of the antibody enzyme conjugate.
(f) The turnover rate of the enzyme used in the conjugate.
(g) The molecular weight and pharmacokinetic of the antibody enzyme conjugate.
(h) The percolation and retention of the antibody enzyme conjugate at the tumour site and its elimination kinetics from normal tissues.
(i) The concentration of free unconjugated endogenous enzyme.
(j) The immunogenicity of the antibody enzyme conjugate.

Despite the obvious complexity of the system, a first clinical trial with a $F(ab')_2$ fragment of a murine anti CEA MAb chemically linked to carboxypeptidase G2 (CPG2) from *Pseudomonas* origin combined with a para-N (mono-2-chloroethyl monomesyl) amino benzoyl glutamic acid prodrug was performed (Bagshawe, 1991). Five patients with advanced colorectal carcinomas were treated. In two out of five patients, regression of CEA expressing metastases was observed in addition to minor responses in three out of five patients. Treatment became inefficient due to the fast activation of a neutralising anti CPG2 immune response. This trial shows that the ADEPT concept works in the patient, but has to be optimised to obtain better therapeutic effects.

Our approach to the ADEPT concept is based on the availability of the high avidity anti CEA MAb BW 431 (Bosslet *et al.*, 1988), its CDR-grafted (Jones *et al.*, 1986; Riechmann *et al.*, 1988) humanised version (Güssow & Seemann, 1991) and the cloned cDNA for human placental β-glucuronidase (Oshima *et al.*, 1987), a lysomal endogenous enzyme (Brot *et al.*, 1978). Out of these human building blocks a fusion gene was constructed (Lorenz *et al.*, 1991). This construction was performed in analogy to the original work of Neuberger's group (Neuberger *et al.*, 1984; Williams & Neuberger, 1986) who showed that a fusion protein between a mouse Fab and nuclease or polymerase could be functionally expressed. In the present paper the molecular and functional characteristics of a fully humanised/human fusion protein expressed in BHK cells will be reported.

### Materials and methods

### *Recombinant DNA-techniques*

Construction and cloning of the fusion gene was done by standard recombinant DNA techniques (Sambrook *et al.*, 1989) as described by Lorenz *et al.* (1991*b*).

### *DNA-transfection*

BHK cells were transfected with plasmid DNA by Ca-phosphate coprecipitation (Graham & van der Eb, 1973). Supernatants of BHK cells growing in selection medium were tested for functionally active fusion protein using the fusion protein activity assay described below.

### *Fusion protein activity assay*

Polystyrol U-bottom microtiter plates were coated with 0.1–0.5 µg CEA or mucin. After blocking with 1% casein in PBS, pH 7.2, 50 µl of BHK transfectoma supernatant were added and removed after 30′ of incubation at RT. After extensive washing of the plates, 50 µl of a 2.5 mM 4-methylumbelliferyl-β-D-glucuronide solution in 200 mM sodium acetate buffer,

Correspondence: K. Bosslet.
Received 15 July 1991; and in revised form 23 October 1991.

pH 5, was added and incubated for 2 h at 37°C. The reaction was stopped using 0.2 M glycine + 0.2% SDS, pH 11.1. Fluorogenic units (FU) were determined as described by Glaser and Sly (1973).

*Avidity determination*

Avidity of fusion protein to purified CEA was determined according to Tipton *et al.* (1990). The avidity to cell bound CEA was measured as described by Bosslet *et al.* (1988).

*Histochemical and immunohistochemical tissue specificity*

Transfectoma supernatants containing fusion protein were added to cryopreserved tissue sections from various human tissues for 30′ at RT. After washing the selectively bound fusion protein was histochemically detected via its enzymatic catalysis resulting in a red unsoluble stain as described by Murray *et al.* (1989) or visualised immunohistochemically using an alkaline phosphatase labelled goat anti β-glucuronidase antibody.

*Purification and analysis of fusion protein*

BHK transfectoma supernatants containing the fusion protein were purified by anti-idiotype affinity chromatography (Bosslet *et al.*, 1991). Adsorption was performed at pH 7.2, elution was done at pH 4. Samples of the elution peak were analysed by SDS-PAGE under reducing or nonreducing conditions followed by silverstaining (Poehling & Neuhoff, 1981) or Western blotting (Towbin & Gordon, 1979).

**Results**

*Short description of the DNA constructs*

A fusion gene was constructed consisting of the human IgG promoter region and signal peptide exon, the humanised version of the rearranged $V_H$ gene of MAb BW 431, the $C_H1$ exon of human IgG3, one hinge exon derived from human $IgG_3$, a synthetic linker peptide and the cDNA coding for the mature human β-glucuronidase. This construct was inserted via its *Hind* III and SalI restriction sites in the pAB-vector (Chart 1) (Zettlmeissl *et al.*, 1987).

A second pAB-vector containing the gene for the humanised light chain of MAb BW 431 is depicted in chart 2. The light chain gene contains the humanised V-gene of the L-chain with IgG promoter region and signal exon and the human C-kappa gene. These two vectors were cotransfected into BHK cells with the plasmid pRMH 140, carrying a neomycin resistance gene (Hudziak *et al.*, 1982) and the plasmid pSVdhfr (Subramani *et al.*, 1981) carrying the dihydrofolate reductase gene allowing positive selection with methotrexate (Wirth *et al.*, 1988).

Chart 1

Chart 2

*Detection of fusion protein secreting transfectomas*

BHK cells were transfected by Ca-phosphate co-precipitation (Graham & van der Eb, 1973) using the four independent plasmids outlined above. Supernatants from transfectoma clones grown in selection medium containing G418 and methotrexate were evaluated in the fusion protein activity assay (see Materials and methods). Out of 97 transfectomas investigated eight transfectomas secreted detectable amounts of molecules with CEA binding potential and β-glucuronidase activity.

*Purification and analysis of fusion protein*

Supernatants from BHK transfectoma cells were processed through an anti-idiotype affinity column as described in Materials and methods. Samples collected from the elution peak at pH 4 were analysed by SDS-PAGE under reducing and nonreducing conditions followed by silverstaining. The SDS-PAGES presented in Figure 1 reveal two bands with an apparent MW of ≈ 100 kDa and ≈ 25 kDa under reducing conditions and two major bands of a MW of ≈ 250 kDa and ≈ 125 kDa under nonreducing conditions. A minor band of a MW of 200 kDa was detected as well.

Western blotting of reduced SDS-PAGE using a horse radish peroxidase labelled (HRP) goat anti human κ antiserum as a detection system revealed a 25 kDa band. Under nonreducing conditions both a 125 kDa and a 250 kDa band were visualised. A 100 kDa band was detected under reducing conditions if a HRP labelled goat anti human β-glucuronidase antiserum was used for immunostaining. Nonreducing conditions revealed two major bands at 125 and 250 kDa after staining with the HRP labelled goat anti human β-glucuronidase antiserum. Furthermore, TSK 3000 gel chromatography of native eluate revealed a single major peak of β-glucuronidase activity at a molecular weight position of approximately 250 kDa (data not shown).

*Avidity determination of the purified fusion protein*

The strength of binding of the purified native fusion protein to purified CEA as well as to cell-associated CEA was determined in comparison to MAb BW 431 or its humanised version. The association constants as determined for the three molecules by two independent methods were in the range of $10^{10}$ l mol$^{-1}$ and did not show significant differences (Table I).

**Figure 1** Photograph showing two silverstained SDS-PAGES. Lanes A represents molecular weight markers. Lane B shows the purified fusion protein under reducing conditions, Lane C under nonreducing conditions.

**Table I** Comparison of the avidity of the murine MAb BW 431 with its CDR-grafted humanised version and the fusion protein

| | $k_a$ ( $\times 10^{10}$ l (Mol)$^{-1}$ | |
|---|---|---|
| mu BW 431 | 3.0–4.7[a] | 3.7[b] |
| hu BW 431 | 1.0–2.2[a] | 1.4[b] |
| hu 431 β-gluc | 1.3–2.0[a] | 2.5[b] |

[a]Tipton et al. (1990); [b]Bosslet et al. (1988).

### Histochemical and immunohistochemical evaluation of the tissue specificity

The avidity evaluation proved that the binding capacity of the fusion protein to purified CEA or CEA bearing cell lines was unchanged compared to the original MAb BW 431 or its humanised version. To further investigate the properties of the fusion protein's binding region we evaluated its histochemical and immunohistochemical specificity on cryopreserved human tissue sections. The data collected from several independent experiments are presented in Table II. Histochemical investigations using naphtyl β-glucuronide combined with hexazotised pararosaniline as substrate for the β-glucuronidase (Murray et al., 1989) revealed that the fusion protein binds to all colon and stomach carcinomas and is enzymatically active. Normal lung and brain are not stained, as expected. Normal liver and kidney are weakly stained due to endogenous lysosomal β-glucuronidase also detectable in the MAb BW 431 negative control (lanes 1 and 2). Furthermore the immunohistochemical specificity using an AP-labelled goat anti human β-glucuronidase antibody or an AP-labelled goat anti mouse Ig antibody showed an identical staining reaction. The colon and stomach carcinomas were positive, whereas the normal tissues like lung, liver, kidney and brain were negative as reported for MAb BW 431. Again a weak reaction due to binding of the anti β-glucuronidase antibody to endogenous β-glucuronidase was observed (lanes 3 and 4). These data clearly demonstrate that the fusion protein has a specificity similar or identical to that of the original MAb BW 431.

### Influence of pH on the catalytic rate of the fusion protein

After the purification of the fusion protein and the determination of its binding potential to purified CEA the turnover rate at various pH values was determined. Figure 2 shows the pH dependence of the fusion protein bound to solid phase attached CEA as revealed by the fusion protein activity assay.

The maximal catalytic rate was observed at pH 4.5. At pH 7.4, the pH in normal tissues, the catalytic rate is about 10% of the optimal value. At pH 7.0, a pH reported for solid tumours (Tannock & Ratin, 1989) the catalytic rate is about 20% of the maximal activity. After adjustment of pH from 7.4 to 4.5 the fusion protein revealed its full catalytic potential arguing for its stability (data not shown).

### Determination of the turnover rate of the fusion protein

With the help of the fusion protein activity assay, a linear correlation between incubation time, fusion protein concentration and liberated fluorogenic units (FU) could be demonstrated (Figure 3). One thousand FU corresponded to 0.14 μg methylumbelliferone (MU), the liberated fluorogenic label. Based on this correlation the turnover rate of the fusion protein was determined. At pH 7.0, 1 Mol of CEA bound

**Figure 2** Fluorogenic units (FU) are plotted against the pH value. Values obtained with 500 ng ——, 83 ng ---- or 16 ng ······ of fusion protein are presented and show a similar type of curve. konz. ——; 1:6 ----; 1:32 ·······. Incubation 2 h of 37°C.

**Table II** Histochemical and immunohistochemical evaluation of the tissue specificity of the fusion protein compared to MAb BW 431

| *Tissues investigated* | *Histochemical substrate reaction using naphtyl β-glucuronide and hexazotised pararosaniline* | | *Immunohistochemical substrate reaction using AP-labelled* — *Goat anti β-glucuronide antibody* | *Immunohistochemical substrate reaction using AP-labelled* — *Goat anti mouse Ig antibody* |
|---|---|---|---|---|
| | fusion protein positive/tested | MAb BW 431 positive/tested | fusion protein positive/tested | MAb BW 431 positive/tested |
| *Carcinomas* | | | | |
| Colon Cas | 7 /7 | 0 /7 | 7 /7 | 7 /7 |
| Stomach Cas | 4 /4 | 0 /4 | 4 /4 | 4 /4 |
| *Normal tissues* | | | | |
| Lung | 0 /3 | 0 /3 | 0 /3 | 0 /3 |
| Liver | 4[a]/4 | 4[a]/4 | 4[a]/4 | 0 /4 |
| Kidney | 2[a]/2 | 2[a]/2 | 2[a]/2 | 0 /2 |
| Brain | 0 /2 | 0 /2 | 0 /2 | 0 /2 |

[a]Endogenous intralysosomal β-glucuronidase detectable in sections without fusion protein also.

fusion protein cleaves ≈ 12,000 moles of methylumbelliferyl β-glucuronide, the synthetic prodrug in 1 h at 37°C.

Similar turnover rates were observed using a prodrug based on daunomycin linked via a urethane nitrophenyl spacer to β-glucuronic acid (manuscript in preparation).

**Figure 3** Fluorogenic units (FU) are plotted against the incubation time at 12.5 ng ——, 2.5 ng ---- or 0.25 ng ······ of fusion protein. A linear time-activity relationship was found at all three fusion protein concentrations.

### Discussion

This study demonstrated that the molecular construct consisting of the binding region of the humanised anti CEA MAb 431 and the human endogenous lysosomal enzyme β-glucuronidase could be expressed and purified to homogeneity as a highly effective fusion protein. Using the 'fusion protein activity assay' we were able to detect eight BHK transfectomas secreting significant amounts of fusion protein that bind to CEA and catalyse the cleavage of the 4-methylumbelliferyl β-glucuronide prodrug to 4-methylumbelliferone and glucuronic acid due to the fusion protein's β-glucuronidase activity. From transfectoma supernatants two molecules could be isolated using anti-idiotype affinity chromatography. Under denaturing conditions, one molecule represented a monovalent protein and the other consisted of a bivalent protein as revealed by our SDS-PAGES and Western blotting data (Figure 1). A schematic diagram of the two molecules is given in Figure 4a. The monovalent molecule (Figure 4b) of a MW of 125 kDa contains the light chain of the humanised MAb BW 431 consisting of the $V_L$ and $C_L$ domains covalently linked by an interchain disulphide bond to the humanised heavy chain. The humanised heavy chain is built up by the $V_H$ and $C_H1$ domain of the humanised MAb BW 431 the N-terminal part of the human $IgG_3$ hinge region, a linker peptide and the human β-glucuronidase. In this monovalent molecule the two hinge region cysteins form an intrachain disulfide bond. If the hinge region cysteins form two interchain disulfide bonds a bivalent fusion protein arises (Figure 4c) which can be isolated from transfectoma supernatants at a similar amount as the monovalent fusion protein (Figure 4c). Under native conditions the two molecular forms exist as bivalent molecules (Figure 4d) as shown by gel chromatography under non denaturing conditions.

Furthermore, the mild elution conditions applicable to the anti-idiotype affinity chromatography resulted in the isolation of a protein fraction with satisfying biochemical properties.

**Figure 4** A schematic diagram of the molecular species of fusion protein secreted from transfectoma supernatant is shown. **a**, Fusion protein molecules found under denaturing conditions after isolation using anti-idiotype affinity chromatography. **b**, Diagram of the fusion protein monomer showing the building blocks of the construct. **c**, Diagram of the fusion protein dimer with covalent linkage by two interheavy chain disulfide bonds. **d**, Diagram of two fusion protein dimers with and without interheavy chain disulfide bonds. Both molecules are found under native conditions after isolation using anti-idiotype affinity chromatography.

Avidity to CEA was found by two independent methods to be in the range of $10^{10}$ l Mol$^{-1}$, a figure similar to the avidity of the murine MAb BW 431 or its humanised version (Table I). Since the molecular forms of the fusion protein as revealed under denaturing conditions are associated in the native state forming molecules with two identical binding sites, the avidity measurements refer to the binding strength of bivalent molecules. In addition, the histochemical and immunohistochemical tissue specificity of the fusion protein was not distinguishable from that of the murine MAb BW 431 (Table II). Not only the binding portion of the fusion protein was functionally active, but also the enzyme moiety showed a catalytic activity and pH profile similar to human β-glucuronidase (Brot et al., 1978). Despite the fact that the catalytic optimum of the fusion protein is at pH 4.5 and the turnover rate at pH 7.0, a pH value assumed for human solid tumours (Tannock & Ratin, 1989), is about 20% of the optimal rate it still seems to be high enough to fulfill the assumptions made in the ADEPT concept. Due to its fully human or humanised building blocks, the fusion protein should have a low immunogenicity in humans, if at all. This characteristic should allow the long term application of this molecule in humans and distinguishes this construct from xenogeneic mouse antibody - bacterial enzyme conjugates which were reported to elicit a fast neutralising antibody response in the patient (Bagshawe et al., 1991). Before the fusion protein can be considered for clinical evaluation, its pharmacodynamic (Natowicz et al., 1979) in relation to the carbohydrate content (Stahl et al., 1976) and therapeutic effects combined with appropriately designed prodrugs (Stella et al., 1985) must be studied in further preclinical model systems. Prodrug systems which are investigated presently are glucuronides of daunomycin and adriamycin. To our knowledge, this is the first report, describing a functionally active fusion protein, consisting of a humanised tumour selective binding portion and a human lysosomal enzyme with potential applicability for a more selective tumour therapy.

The skillful technical assistance of Holger Lind, Gerd Lauer, Norbert Döring, Dieter Busch, Hartmut Schmidt and Christine Wetzler are greatly appreciated. Furthermore, we thank Mrs Sylvia Lehnert for her dedicated secretarial assistance.

The cDNA coding for human β-glucuronidase was a generous gift of Drs Sly and J.W. Kyle from St Louis, University School of Medicine.

### References

BAGSHAWE, K.D. (1987). Antibody directed enzymes revive anti-cancer prodrugs concept. *Br. J. Cancer*, **56**, 531.

BAGSHAWE, K.D., SPRINGER, C.J., SEARLE, F. & 4 others (1988). A cytotoxic agent can be generated selectively at cancer sites. *Br. J. Cancer*, **58**, 700.

BAGSHAWE, K.D., SHARMA, S.K., ANTONIW, P. & 5 others (1991). Antibody directed enzyme prodrug therapy (ADEPT). First clinical report. In: *Antibody Immunoconjugates and Radiopharmaceuticals*. Vol. 4, 2; Orders, S.E. (ed.). M.A. Liebert Inc: New York, p. 204, Abstract 0-11.

BOSSLET, K., STEINSTRAESSER, A., SCHWARZ, A. & 4 others (1988). Quantitative considerations supporting the irrelevance of circulating serum CEA for the immunoscintigraphic visualization of CEA expressing carcinomas. *Eur. J. Nucl. Med.*, **14**, 523.

BOSSLET, K., STEINSTRAESSER, A., HERMENTIN, P. & 6 others (1991). Generation of bispecific monoclonal antibodies for two phase radioimmunotherapy. *Br. J. Cancer*, **63**, 681.

BROT, F.E., BELL, C.E. & SLY, W.S. (1978). Purification and properties of β-glucuronidase from human placenta. *Biochemistry*, **17**, 385.

GLASER, J.H. & SLY, W.S. (1973). *J. Lab. Clin. Med.*, **82**, 969.

GRAHAM, F.L. & EB, A.J. VAN DER (1973). A new technique for the assay of infectivity of human adenovirus 5 DNA. *Virology*, **52**, 456.

GÜSSOW, D. & SEEMANN, G. (1991). Humanisation of monoclonal antibodies. *Methods of Enzymol.* (in press).

HUDZIAK, R.M., LASKI, F.A., RAJBHANDARY, U.L., SHARP, P.A. & CAPECCHI, M.R. (1982). Establishment of mammalian cell lines containing multiple nonsense mutations and functional suppressor tRNA genes. *Cell*, **31**, 137.

JONES, P.T., DEAR, P.H., FOOTE, J., NEUBERGER, M.S. & WINTER, G. (1986). Replacing the complementarity-determining regions in a human antibody with those from mouse. *Nature*, **321**, 522.

LORENZ, P., SCHUERMANN, M. & SEEMANN, G. (1991). Construction and expression of an antibody/enzyme fusion molecule for antibody dependent enzyme prodrug therapy (ADEPT). *J. Cancer Res. Clin. Oncol.*, p. 9, Abstract I6.

LORENZ, P. (1991). *Konstruktion und Expression von rekombinanten Antikörper-Enzym-Hybridmolekülen für die Tumortherapie*. Diplomarbeit, At the Faculty for Medicine at the Philipps-University, Marburg.

MURRAY, G.I., BURKE, M.D., & EWEN, S.W.B. (1989). Enzyme histochemistry on freeze-dried, resin-embedded tissue. *J. Histochem. Cytochem.*, **37**, 643.

NATOWICZ, M.R., CHI, M.M.-Y., LOWRY, O.H. & SLY, W.S. (1979). Enzymatic identification of mannose 6-phosphate on the recognition marker for receptor-mediated pinocytosis of β-glucuronidase by human fibroblasts. *Proc. Natl Acad. Sci. USA*, **76**, 4322.

NEUBERGER, M.S., WILLIAMS, G.T. & FOX, R.O. (1984). Recombinant antibodies possessing novel effector functions. *Nature*, **312**, 604.

OSHIMA, A., KYLE, J.W., MILLER, R.D. & 7 others (1987). Cloning, sequencing and expression of cDNA for human β-glucuronidase. *Proc. Natl Acad. Sci. USA*, **84**, 685.

PHILPOTT, G.W., BOWER, R.J. & PARKER, C.W. (1973*a*). Selective iodination and cytotoxicity of tumor cells with an antibody-enzyme conjugate. *Surgery*, **74**, 51.

PHILPOTT, G.W., SHEARER, W.T., BOWER, R.J. & PARKER, C.W. (1973*b*). Selectivity cytotoxicity of hapten-substituted cells with an antibody-enzyme conjugate. *J. Immunol.*, **111**, 921.

PHILPOTT, G.W., BOWER, R.J., PARKER, K.L., SHEARER, W.T. & PARKER, C.W. (1974). Affinity cytotoxicity of tumor cells with antibody-glucose oxidase conjugates, peroxidase and arsphenamine. *Cancer Res.*, **34**, 2159.

POEHLING, H.-M. & NEUHOFF, V. (1981). Visualization of proteins with a silver 'stain': a critical analysis. *Electrophoresis*, **2**, 141.

RIECHMANN, L., CLARK, M., WALDMANN, H. & WINTER, G. (1988). Reshaping human antibodies for therapy. *Nature*, **332**, 323.

SAMBROOK, J., FRITSCH, E.F. & MANIATIS, T. (1989). *Molecular Cloning: a Laboratory Manual*. Cold Spring Harbor Laboratory Press.

SHARMA, S.K., BAGSHAWE, K.D., BURKE, P.J., BODEN, R.W. & ROGERS, G.T. (1990). Inactivation and clearance of an anti-CEA carboxypeptidase G2 conjugate in blood after localisation in a xenograft model. *Br. J. Cancer*, **61**, 659.

SUBRAMANI, S., MULLIGAN, R. & BERG, P. (1981). Expression of the mouse dihydrofolate reductase complementary deoxyribonucleic acid in simian virus 40 vectors. *Mol. Cel. Biol.*, **1**, 854.

STAHL, Ph., SIX, H., RODMAN, J.S., SCHLESINGER, P., TULSIANI, D.R.P. & TOUSTER, O. (1976). Evidence for specific recognition sites mediating clearance of lysosomal enzymes *in vivo*. *Proc. Natl Acad. Sci. USA*, **73**, 4045.

STELLA, V.J., CHARMAN, W.N.A. & NARINGREKAR, U.H. (1985). Prodrugs. Do they have advantages in clinical practice? *Drugs*, **29**, 455.

TANNOCK, I.F. & RATIN, P. (1989). Acid pH in tumors and its potential for therapeutic exploitation. *Cancer Res.*, **49**, 4373.

TIPTON, D.A., WALTER, W.S. & SCHONBAUM, G.R. (1990). Epitope mapping of horse radish peroxidase with use of monoclonal antibodies. *Hybridoma*, **9**, 319.

TOWBIN, T. & GORDON, J. (1979). Electrophoretic transfer of proteins from polyacrylamide gels to nitrocellulose sheets: procedure and some applications. *Proc. Natl Acad. Sci. USA*, **76**, 4350.

WILLIAMS, G.T. & NEUBERGER, M.S. (1986). Production of antibody-tagged enzymes by myeloma cells: application to DNA polymerase I Klenow fragment. *Gene*, **43**, 319.

WIRTH, M., BODE, J., ZETTLMEISSL, G. & HAUSER, H. (1988). Isolation of overproducing recombinant mammalian cell lines by a fast and simple selection procedure. *Gene*, **73**, 419.

ZETTLMEISSL, G., RAGG, H. & KARGES, H.E. (1987). Expression of biologically active human antithrombin III in Chinese hamster ovary cells. *Bio/Technology*, **5**, 720.